# Task Transfer Learning for EEG Classification in Motor Imagery-Based BCI System

**DOI:** 10.1155/2020/6056383

**Published:** 2020-12-15

**Authors:** Xuanci Zheng, Jie Li, Hongfei Ji, Lili Duan, Maozhen Li, Zilong Pang, Jie Zhuang, Lu Rongrong, Gao Tianhao

**Affiliations:** ^1^College of Electronics and Information Engineering, Tongji University, Shanghai 201804, China; ^2^Department of Electronic and Computer Engineering, Brunel University London, Uxbridge UB8 3PH, UK; ^3^School of Psychology, Shanghai University of Sport, Shanghai 200438, China; ^4^Department of Rehabilitation, Huashan Hospital, Fudan University, Shanghai 200040, China

## Abstract

The motor-imagery brain-computer interface system (MI-BCI) has a board prospect for development. However, long calibration time and lack of enough MI commands limit its use in practice. In order to enlarge the command set, we add the combinations of traditional MI commands as new commands into the command set. We also design an algorithm based on transfer learning so as to decrease the calibration time for collecting EEG signal and training model. We create feature extractor based on data from traditional commands and transfer patterns through the data from new commands. Through the comparison of the average accuracy between our algorithm and traditional algorithms and the visualization of spatial patterns in our algorithm, we find that the accuracy of our algorithm is much higher than traditional algorithms, especially as for the low-quality datasets. Besides, the visualization of spatial patterns is meaningful. The algorithm based on transfer learning takes the advantage of the information from source data. We enlarge the command set while shortening the calibration time, which is of significant importance to the MI-BCI application.

## 1. Introduction

As a technique decoding brain activity, Brain-Computer Interface (BCI) based on electroencephalogram (EEG) enables people to interact with computers without the involvement of peripheral muscular activity, which builds a communication bridge between the brain and computer. With signal processing, pattern classification, machine learning, and other techniques, the BCI system translates different kinds of brain activities such as attentive mental states [1], motor imagery [2–5] (MI), and so on into machine instruction for controlling devices. For example, it could be used to control unmanned aerial vehicles [5], to help train solders [6], to help patients with motor disabilities, such as amyotrophic lateral sclerosis [7], brainstem stroke [2, 8] to recover.

Among most studies about EEG signal classification, researchers are highly concerned about the EEG signal generated from motor imagery which has been widely used in many BCI applications [2–5]. Motor imagery is a BCI paradigm in which brain activity is generated at the sensorimotor cortex during the imagination of the limb movement [9] such as left hand (LH), right hand (RH), and both feet (F). During the MI experiment, the power of the alpha band (8-12 Hz) and beta band (13-30 Hz) increase or decrease in the sensorimotor cortex of the ipsilateral hemisphere and the contralateral hemisphere [10, 11]. The power suppression and enhancement observed through EEG signal are, respectively, called event-related desynchronization (ERD) and event-related synchronization (ERS) [12]. The ERD/ERS patterns can be used for translating brain activity and classifying the imagination of limbs through machine learning.

However, most of MI-BCI promising applications are prototypes and still scarcely used outside laboratories. Among the reasons preventing MI-BCI from being widely put into practice, we want to give solutions to two of them, which are the long calibration time [13–16] and the lack of enough MI commands [17].

The first problem, namely, the long calibration time, means researchers require a large number of calibration trials for training a subject-specific and task-specific model, which is time-consuming in collecting data and training model. Due to the intersubject and intertask variability of the EEG signal [18], the training samples need to contain the EEG data from every MI commands for model construction. Thus, the data collection for BCI takes a lot of time. And the variability between datasets makes it difficult to give a model fit for every subject, which lengthens the time for the training model. As for the second question, the lack of enough MI commands makes it less available for BCIs. In the traditional MI experiment, there are almost 4 types of commands for subjects to imagine, that is left hand, right hand, both feet, and tongue [19].

In order to add more commands into the BCI system, the combination of single limbs command can be added into the command set. For example, on the basis of existing MI command (LH, RH, and F), we can obtain combined commands, movement of both left hand and right hand simultaneously (LH&RH), movement of the left hand and both feet simultaneously (LH&F), and movement of the right hand and both feet simultaneously (RH&F). However, researchers have to collect the same scale of dataset for new MI commands as which for old commands, which multiples the time spent on data collection. The larger MI dataset also lengthen the time spent on model training. Besides, as for a new subject, the imagination of new MI commands is harder than typical commands, which leads them to train those commands for a long period of time before the recording. Due to the increasing number and complexity of the entire six commands, the expansion of the command set becomes less feasible.

In order to shorten the time on model construction as well as enlarge the command set, transfer learning would have a profound impact. Transfer learning algorithms use datasets, features, or model parameters [20] from the source domain for training the model in the target domain so as to reduce the scale of training data in the target domain, which reduces the sampling and training cost. In the classification of the EEG signal, there have been many algorithms based on task-to-task transfer and subject-to-subject transfer in response to intertask and intersubject variability. Among those algorithms, the features extractors based on the Common Spatial Pattern algorithm (CSP) are one of the mainstream techniques. The method of CSP designs spatial filters, which maximize the discriminability of two classes of data and make the variances in filtered data are optimal for classification [21–23]. Based on CSP and transfer learning, researchers raised a lot of algorithms to generate spatial filters from the source domain, for example, Regularized CSP [24–26], stationary subspace CSP [27], Bayesian CSP [28]. Through adjusting parameters, finding the common subspace, and using the Bayesian model, similar features from source subjects can be shared and the feature extractors from the source domain can be used in the target domain.

The solution to calibration time extension caused by command set expansion is to decrease the scale of the dataset collected for new commands. Therefore, in order to increase the number of commands on the premise of not lengthening calibration time, it is proper to make the most of other available data for new filters and classifiers. While the intertask variability of EEG signals varies from one class to another, the principle feature characteristics remain invariant across classes. Thus, although it is unwise to simply add training set of old commands to that of new classes, the prior knowledge collected from the previous training set can be used into the building of filters and classifiers for new classes. Consequently, using transfer learning to construct model is an optimal solution. We regard data from typical commands and new commands as source data and target data, respectively. And we use source data to improve the property of our model for target data.

In this paper, we provide a spatial pattern transfer algorithm. We add a screening process before the utilization of spatial filters generated from datasets of old MI commands. In order to shrink the difference of the generated filters and objective filters, we reject the filters performing badly based on the fisher ratio [29, 30].

The remainder of this paper is structured as follows. In Section 2, we introduce our experimental paradigm, our dataset as well as our algorithm. In Section 3, experimental results are shown. The paper concludes with a discussion of the results in Section 4.

## 2. Materials and Methods

In this section, we show our experiment and our algorithm used for the data recorded in the experiment.

### 2.1. Experiment and Data Processing

#### 2.1.1. Experiment

We recorded brain activity from 5 healthy subjects in the experiment. The BCI paradigm consisted of six different motor imagery tasks, namely the imagination of the left hand (class 1), right hand (class 2), both feet (class 3), left hand and right hand (class 4), left hand and both feet (class 5), and right hand and both feet (class 6). On the basis of traditional commands, subjects were more familiar with the combined commands comparing to imagining new body parts. Consequently, we chose LH&F, RH&F, and LH&RH as our new commands.

The experiment was comprised of 6 runs separated by short breaks. One run consisted of 60 trials (10 for each of the six classes), yielding a total of 360 trials for every subject (60 trials in each class).

**Figure 1:**
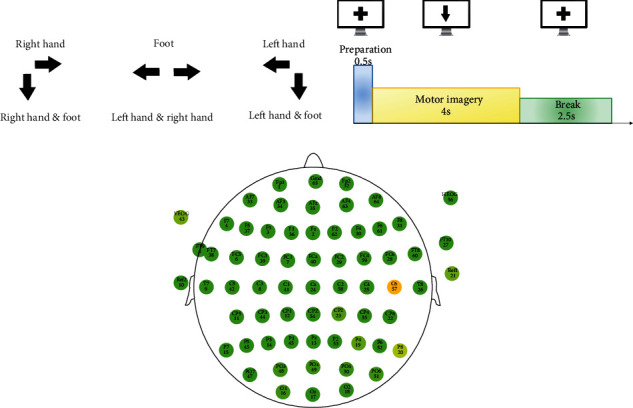
(a) The correspondence between the image on the screen and task of MI commands. (b) The time series of one trial in a run, and the image shown on the screen. (c) Electrode montage was used in the experiment.

The subjects in this experiment sat in a comfortable chair in front of a computer screen. At the beginning of a trial, a fixation cross appeared on the white screen. After 500 ms, an image in the form of one or two arrows pointing different directions corresponding to one of six classes appeared and stayed on the screen for 4 s. The relation between images and MI commands are showed in Figure 1(a). The image prompted the subjects to perform the MI task as requested until the fixation cross appeared again after the appearance of the image. The subject had a short break for 2.5 s and waited for the next trial. The paradigm is illustrated in Figure 1(b).

Multichannel EEG amplifiers with 64 channels band-pass filtered between 0.05 and 200 Hz and sampled at 500 Hz were used to record the EEG, whose montage is shown in Figure 1(c). Horizontal and vertical EOG signals were recorded to check for eye movements, which were not used for classification.

#### 2.1.2. Preprocessing

During the process of preprocessing, we removed noise caused by eye and muscle movement and select effective rhythm (sensorimotor rhythm) in order to enhance the relevant information. Initially, we rereferenced the data. The left mastoid was chosen as reference electrode during the collection. In order to avoid laterality bias in the data, we rereferenced the data offline by changing the reference electrodes into the average of value of the left and right mastoids. We removed contamination from bad channels by using the average of channels around the bad channel to replace them. Then, the whole time series of EEG data was band-pass filtered in 8-30 Hz, as written in [23]. We ran ICA to reject the EOG artifacts. We extracted epochs from the whole time segment located from 0.5 s before instructing the subject to perform MI to 2 s after instructing. Signals were baseline corrected over the interval 0-500 ms before instructing. Finally, the processed data was visually screened to discard any noise trials.

### 2.2. Method

In this subsection, we show the total scheme of our algorithm and detailed description of every essential component.

#### 2.2.1. The Total Scheme of the Algorithm

**Figure 2:**
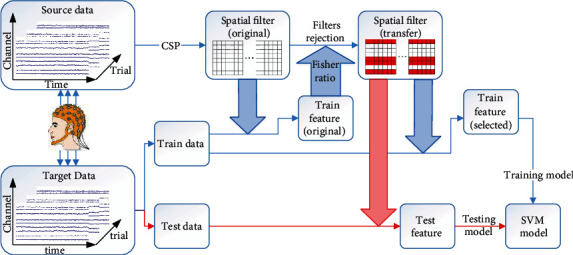
The total structure of our algorithm. The blue line stands for the training process, and the red line stands for the testing processing.

The total scheme of the algorithm is organized as described in Figure 2, which can be divided into model training and model test. The training of the model contains four components. First, the EEG signal is collected during the motor imaginary experiment, preprocessed, and divided into source data and target data according to our standard. Second, the original spatial patterns are constructed from source data based on the CSP algorithm. Third, with the fisher ratio algorithm, we transfer the original spatial filters into spatial filters for target data. At last, features extracted by filters are given as input to a Support Vector Machine (SVM) classifier [31, 32] for training the model. After the model has been trained, it can be used for the test data. The features of test data are also extracted by transfer spatial filters. And those features are used as input of classifier for the testing model.

#### 2.2.2. Data Transfer Standard on Division and Selection

The preprocessed data will be divided into source data and target data. The source data comes from the old command, and target data from the new command. In order to improve the stability of the transfer model, we need to choose proper source data with the right label. In this section, we give our standard of choosing source data and the reason why we select those data depending on the target data. Initially, because of the intersubject variability of the spatial features between each subject, we select source data and target data from the same subject. And owing to the domain adaptation [18] standard in the transfer learning area, we choose the most similar data between source data and target data in terms of the imagery of body parts. For example, as for the classification of LH&F and RH&F, we use the dataset from LH and RH as source data. As for the classification of LH&RH and RH&F, we use the dataset from F and RH as source data. The standard for data selection will also be discussed in Section 4.4.

#### 2.2.3. Original Spatial Pattern Construction Based on CSP

As for the classification of two distributions in a high-dimensional space, the CSP algorithm designs spatial filters, which maximize variance for one class and simultaneously minimize variance for the other class. Based on the simultaneous diagonalization of covariance matrices of two classes, spatial filters can lead to features whose variances are optimal for the discrimination.

In the traditional algorithm, in order to construct spatial filters extracting optimal features, the scale of training data is always large. By using source data, we can decrease the dependence on the training data. In order to make the most of the source data, we use source data for constructing the spatial filters. In the following section, we will take a particular classification as an example. We will use the source data (LH and RH) to construct the source spatial filters for the classification of target data (LH&F and RH&F).

The single-trial EEG signal from source data is represented as *N* × *G* matrix X_c_^*i*^. *N* represents the number of channels, and *G* is the number of samples per channel. *X*_*c*_^*i*^ is the *i*^th^ (*i* ∈ [1, *K*]) trial of EEG signal matrix which belongs to the class c (*c* ∈ {1, 2}). The class 1 refers to data from LH, and the class 2 refers to RH. The average spatial covariance matrix of each class can be calculated as

(1) $$\begin{array}{c} R_{c}=\frac{1}{K}\underset{i}{\sum }\frac{X_{c}^{i}{\left(X_{c}^{i}\right)}^{T}}{\text{trace}\left(X_{c}^{i}{\left(X_{c}^{i}\right)}^{T}\right)}. \end{array}$$

*R* is the sum of the covariance matrix from source data. And the composite spatial covariance matrix *R* can be factorized as

(2) $$\begin{array}{c} R=R_{1}+R_{2}=U\Lambda U^{T}. \end{array}$$

*U* is the matrix of eigenvectors, and *Λ* is the diagonal matrix of corresponding eigenvalues. Then, the whitening transformation is obtained as

(3) $$\begin{array}{c} P=\sqrt{\Lambda^{-1}}U^{T}. \end{array}$$

*R*_1_ and *R*_2_ from LH and RH are whitened as *S*_1_ and *S*_2._

(4) $$\begin{array}{c} S_{1}=PR_{1}P^{T},S_{2}={PR}_{2}P^{T}. \end{array}$$

Then, *S*_1_ and *S*_2_ can be factorized as

(5) $$\begin{array}{c} S_{1}=B\Lambda_{1}B^{T},S_{2}=B\Lambda_{2}{\text{B}}^{\text{T}},\Lambda_{1}+\Lambda_{2}=\text{I} \end{array}$$

*Λ*_1_ and *Λ*_2_ are diagonal matrices. *I* is the identity matrix, and *S*_1_ and *S*_2_ share the same eigenvector. Therefore, this property makes the eigenvectors B effective for the discrimination of two classes. $\hat{B}$ is the matrix of the *m* first and last eigenvectors in *B*. The spatial filters *ω* can be calculated as

(6) $$\begin{array}{c} \omega ={\overset{\wedge }{B}}^{T}P. \end{array}$$

#### 2.2.4. Pattern Transfer Based on Fisher Ratio

The original spatial filters created from source data are in a large scale. There is no doubt that the original spatial filters are suitable for source data. However, not all filters can be used to extract features for target data. We need to select a valuable subset of filters from the original filters so as to transfer the source filters into target filters. In this section, we briefly introduce pattern transfer based on fisher ratio. The filters we need can project target data into optimal features which have higher fisher ratio. The purpose of the fisher ratio is to find a subset of features, in which the distances between each data in different classes are as large as possible, while the distances between each data in the same class are as small as possible. Therefore, the fisher ratio is defined as the ratio of the variance between classes to the variance within classes. There is a dataset *D* which contains *n* samples (*ω*_1_, *ω*_2_, ⋯, *ω*_*n*_), which belongs to C classes. Each of the sample *ω* has *K* features (*x*^*k*^, *k* ∈ [1, *K*]). There are *n*_*i*_ samples in one class. *m*_*i*_^*k*^ is the mean value of *x*^*k*^ in one class. And *m*^*k*^ is the mean value of all *x*^*k*^. Thus, the fisher ratio of the features in every dimensionality can be calculated as

(7) $$\begin{array}{c} J_{k}=\frac{S_{\text{between}}^{k}}{S_{\text{within}}^{k}},\\ S_{\text{between}}^{k}=\overset{C}{\underset{i=1}{\sum }}\frac{n_{i}}{n}{\left(m_{i}^{k}-m^{k}\right)}^{2},\\ {\text{S}}_{\text{within}}^{\text{k}}=\frac{1}{n}\overset{C}{\underset{i=1}{\sum }}\underset{x\in \omega_{i}}{\sum }{\left(x^{k}-m_{i}^{k}\right)}^{2}. \end{array}$$

The higher the fisher ratio of the feature is, the more discriminative the features between the two classes are.

**Figure 3:**
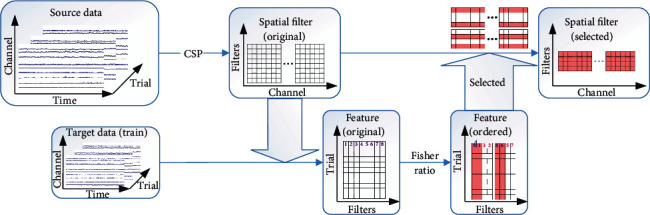
The transfer of spatial patterns based on fisher ratio. The features which are colored in pink refer to features in higher fisher ratio. And the filters in pink refer to selected filters.

Getting back to the pattern transfer, we think that the more optimal filter is, the higher the fisher ratio of feature is extracted. Consequently, to test the performance of each line of filters, we sort features generated through the projection of train data on original spatial filters. The score of each dimensionality in features calculated by the fisher ratio algorithm represents the performance of each spatial filter. Based on the principle of CSP, the original filters from source data should be divided into two groups which gives the larger eigenvalues of *S*_1_ and smaller eigenvalues of *S*_2_, respectively. Consequently, the transfer filters should also be selected by the same amount in each group. We divide the original spatial filters into two equal groups, which, respectively, are generated from the first and last eigenvectors through CSP. Then, we select the same amount of filters, which have a higher score in each group, and combine them as transfer filters. The transfer of filters is described as Figure 3

#### 2.2.5. SVM for Classification

Before the classification, we use our transfer spatial filters for extracting features. With the transfer spatial filters *ω*′, single-trial EEG signal *X*_*c*_^*i*^ can be calculated as

(8) $$\begin{array}{c} Z=\omega^{\prime }X_{c}^{i}. \end{array}$$

The feature vector *f*_*p*_^*i*^ can be calculated as

(9) $$\begin{array}{c} f_{p}^{i}=\log\left(\frac{\text{va}{\text{r}}_{p}^{i}}{\sum_{p=1}^{2m}\text{v}\text{a}{\text{r}}_{p}^{i}}\right). \end{array}$$

var_*p*_^*i*^ is the variance of *p*^th^ row in *Z* matrix.

Aiming at finding decision boundary between the class samples, SVM is an algorithm which can effectively prevent the defects of traditional classification algorithm, such as overtraining, dimension disaster, and local minima [31, 33]. The SVM classifier maximizes the distance between decision boundary and margin. Due to the strength of the SVM classifier, it has been widely used in BCI classification [34, 35].

We utilized the entire 60 trials of source data from two selected classes which have been introduced in the previous section and 5 trials each class of target data for constructing spatial filters. In the selection of filters based on the fisher ratio, we chose the top 4 in the original filters which have higher scores. Then, we transferred the previous 5 trial target data into training features with the selected spatial filter. Combining with corresponding class information as the training label, we trained the classifier model.

As for the SVM classifier, the Gaussian radial basis function is used as the kernel function, and a five-hold cross-validation is used to choose suitable parameters for the testing data.

## 3. Results and Discussion

The performance of our algorithm in our dataset is compared with two popular algorithms, which are CSP and power spectrum density (PSD) [36]. We utilize each algorithm to extract features from the testing set of the target data. In the CSP algorithm, we use spatial filters generated from target data. As for PSD, we use power spectral density values from one-way EEG data at particular channels (C3, C4, and Cz). We also calculate averaged PSD for the three channels. In order to test the robustness of the algorithm, we use the 12-CV 5 times on the target dataset of each subject. In order to decrease the scale of transfer data, in the cross-validation, we use 1/12 as training data and 11/12 as test data. We will compare the average accuracy of all algorithms. And the comparison of the different discriminative patterns from different algorithms will be showed in the following part. Besides, in order to make the comparison more particularly, the spatial patterns have been illustrated by focusing on the particular brain region.

### 3.1. PSD Analysis for the Dataset

**Figure 4:**
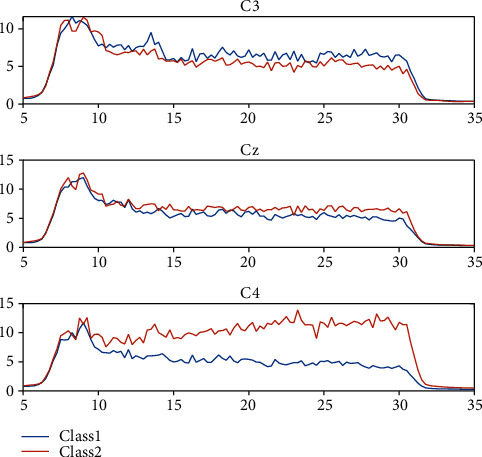
PSD analysis for imagining LH&F (class1) and RH&F (class2). The noticeable difference can be observed around 10-30 Hz in the average power spectrum at C3, C4, and Cz for two performed tasks. The blue line stands for the imagery of LH&F, and the red line stands for the imagery of RH&F.

In order to test the availability of the EEG dataset, PSD features at particular channels in the motor cortex (C3, C4, and Cz) are calculated based on temporal Fourier transform for each trial of EEG data. Noticeable differences can be observed in the averaged PSD for two target tasks. Imagining the LH&F movement leads to the decrease of alpha and beta bands' power at C4 and Cz channels and the increase at C3, whereas the contrary phenomena occur during imagining RH&F movement. The phenomenon from PSD analysis confirms the description of ERD/ERS.  Figure 4 shows the average power spectrum at channel C3, Cz, and C4 for 3rd subject, evident differences are presented between the two tasks, especially around 10-30 Hz.

### 3.2. Spatial Patterns Illustration

**Figure 5:**
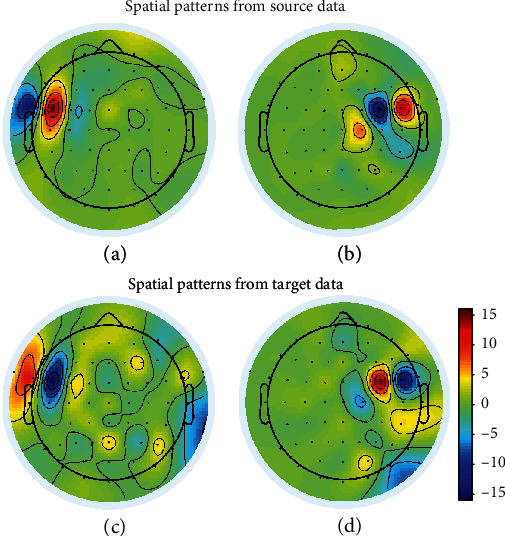
Spatial patterns of two most significant features in the transfer CSP and CSP algorithms, which were extracted from the 3rd subject's imagery of LH&F and RH&F. (a, c) are spatial patterns of LH&F and (b, d) are spatial patterns of RH&F.

**Figure 6:**
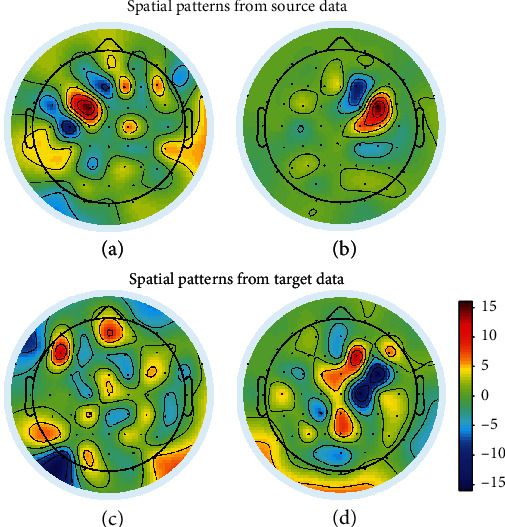
Spatial patterns of two most significant features in the transfer CSP and CSP algorithms, which were extracted from 1st subject's imagery of LH&F and RH&F. (a, c) are spatial patterns of LH&F and (b, d) are spatial patterns of RH&F.

In order to show the superiority of transfer spatial patterns, we visualize the spatial patterns from our algorithm and traditional CSP algorithm and compare the visualization of them. The visualization of the two most discriminative spatial patterns extracted through two algorithms from the 3rd and 1st subjects is illustrated in Figures 5 and 6. In both figures, the spatial patterns in the first row are generated from our algorithm, and the spatial patterns in the second row are generated from CSP. As for the two subjects, the classification accuracies of 3rd subject for both algorithms is higher than the 1st subject. The accuracies for the 3rd subject in our algorithm and CSP are, respectively, 99.09% and 98.18%, which has a high accuracy in both algorithms and the optimization of the algorithm can only cause a little increase. As for the 1st subject, the accuracies are, respectively, 69.17% and 56.67%, which has a lower accuracy in the CSP algorithm but has a great increase in accuracy caused by the transfer algorithm. In Figure 5, the visualization shows that the highest discriminative weights can be viewed at FC5, FC6 channels from both patterns of our algorithm and CSP. The physiologically evidences from both our algorithm and CSP are similar to the result showed in PSD analysis. In Figure 6, the visualization shows that the patterns from our algorithm are clearer than patterns in CSP, which is fitter for the result in PSD comparing to the traditional algorithm. The optimization in spatial pattern also causes the increase in classification.

### 3.3. Classification Results on the Testing Set

**Figure 7:**
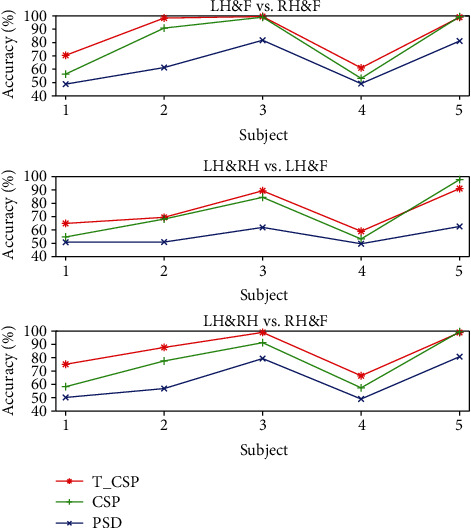
The average accuracy of classification on three different tasks (LH&F vs. RH&F, LH&RH vs. LH&F, and LH&RH vs. RH&F). The different color of lines refers to different algorithms. The *x*-axis refers to the number of subjects.

**Figure 8:**
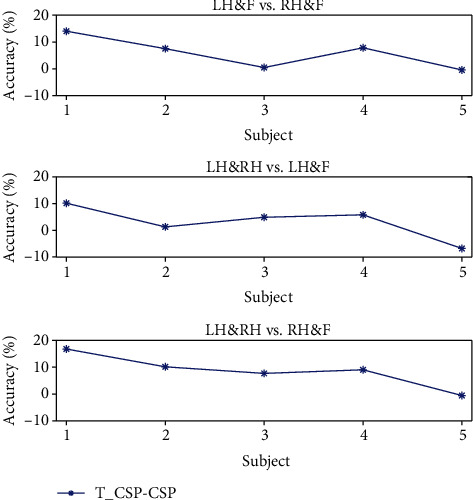
The difference of accuracy between our algorithm and CSP algorithm, the increase of accuracy performs remarkably in the 1st and 4th subjects whose data quality relatively worse in all of the subjects.

The average classification results for all algorithms are listed in Table 1 and showed in Figure 7. As for every task of every subject, we run 12-CV for 5 times and collect the average accuracy and the range of the accuracy. Because of the different quality of data from each subject, the average accuracy from each subject in each task varies in a large range. However, we find that our algorithm performs better than two baseline algorithms on most subjects. In addition, it performs much better as for the dataset which performs badly in motor imagery tasks. Based on the accuracy in traditional algorithms, we find that the accuracies of the 1st and 4th subjects are relatively lower in all of the accuracies, which means the 1st and 4th subjects perform worse in all of the subjects. The difference on the accuracy between our algorithm and traditional algorithm is shown in Figure 8. We can find that the increase on accuracy of the 1st and 4th is more remarkable compared to all of the subjects.

### 3.4. Classification Results on Different Task

**Figure 9:**
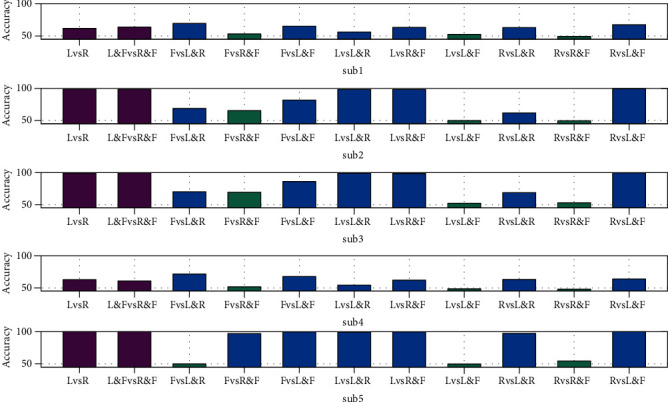
The accuracy of classification on three different kinds of tasks (both old commands, both new commands, new commands and old commands) using the same source data. The *x*-axis refers to different groups of tasks. The *y*-axis refers to accuracy. The bars in purple mean the first and second kinds of classification, and the rest refers to the third group. The green bars mean classification which has lower accuracy.

In order to check the performance of the algorithm when classifying different tasks, we use the spatial pattern transfer algorithm on different command tasks. We use data from LH and RH as source data for three different kinds of target data classification, which are target data from old commands, new commands, and both old and new commands. Aiming at the source data, we choose the LH vs. RH as the first group, and LH&F vs. RH&F as the second group. As for the third group which is the classification between old command and new command, we make the classification for each two groups which means LH vs. LH&F, LH vs. RH&F, LH vs. LH&RH, RH vs. LH&F, RH vs. RH&F, RH vs. LH&RH, F vs. LH&F, F vs. RH&F, and F vs. LH&RH. The classification results are listed in Table 2 and showed in Figure 9. Comparing the accuracy of all tasks using the same source data, we find that our spatial filters can classify both old commands and both new commands in a similar accuracy. However, as for the classification between new commands and old commands, the accuracy is seriously influenced by the similarity of the MI tasks. According to the comparison of accuracy in the second group, we find that each of the new commands has one type of old commands which is really similar and should be used as source data for the classification of new commands. For example, in the comparison of the LH vs. LH&F, RH vs. LH&F, and F vs. LH&F, we find that LH vs. LH&F caused a really low accuracy which contrast the accuracy of RH vs. LH&F and F vs. LH&F.

## 4. Conclusion

In order to add more MI commands, we add the combination of traditional MI commands into the BCI system. To reduce the time for researchers spending on collecting data and training model for new commands, this paper presented a new algorithm for feature extracting based on transfer learning. On one hand, it reduces the long calibration time through making the most of existing data and decreasing the number of training data in new MI commands. On the other hand, it increases the accuracy of the classification on less training samples in the BCI system. Our algorithm solves the problem of long calibration time and lack of MI commands, as well as increases the accuracy. Furthermore, because of its outstandingly increasing performance in subjects who have low accuracy in traditional CSP classification, it is helpful for subjects who cannot perform well in the BCI system. Our algorithm makes the BCI system more user-friendly for subjects. There is no need for subjects to train for a long time in order to adapt the MI tasks.

We compared our algorithm and traditional algorithms on the dataset collected by our laboratory. The spatial patterns from our algorithm are more physiologically reasonable in bad performing subjects. And our algorithm performs better than traditional algorithms.

In summary, our algorithm is better than traditional algorithms especially for subjects with less training samples and poor performances in traditional algorithm. With good performance and stability, our proposed algorithm can reduce the need for the training samples of new MI command by transferring the source data from the old MI command.

The method of spatial patterns is suitable for discriminating between 2 classes. Through changing the multiclass classification into binary classes, our algorithm can also be used in multiclass problem. In the future, with more state-of-the-art feature extractor from our algorithm, we can utilize it on feature extraction of deep transfer learning. With the help of a deep learning algorithm and the optimal features, the performance of classification may become better.

## Figures and Tables

**Table 1:** The average classification accuracy and accuracy range of three algorithms on the target dataset.

	Average accuracy (max-min)		
	Transfer CSP	CSP	PSD
Sub 1	70.13% (79.58%-66.25%)	56.47% (70.83%-48.33%)	49.95% (56.25%-40.42%)
Sub 2	85.18% (91.43%-69.05%)	78.85% (92.38%-63.81%)	56.35% (70.00%-39.05%)
Sub 3	95.93% (98.79%-81.52%)	91.57% (99.39%-74.24%)	74.32% (88.79%-46.06%)
Sub 4	62.16% (78.61%-42.50%)	54.60% (69.72%-42.50%)	49.31% (56.39%-42.22%)
Sub 5	96.29% (99.39%-84.85%)	98.86% (100.00%-96.36%)	74.83% (91.82%-51.52%)

**Table 2:** The accuracy of classification on three different kinds of tasks (both old commands, both new commands, new commands and old commands) using the same source data.

Subject	LvsR	L&FvsR&F	FvsL&R	FvsR&F	FvsL&F	LvsL&R	LvsR&F	LvsL&F	RvsL&R	RvsR&F	RvsL&F
Sub1	61.9%	63.9%	69.5%	53.2%	65.1%	56.0%	63.4%	52.4%	63.2%	49.3%	67.6%
Sub2	98.6%	98.6%	68.9%	65.4%	81.9%	98.3%	98.5%	49.9%	61.9%	49.6%	99.6%
Sub3	99.1%	99.6%	70.2%	69.5%	86.3%	98.9%	98.7%	52.3%	69.0%	53.0%	99.8%
Sub4	63.1%	61.0%	71.9%	51.8%	67.9%	54.5%	62.2%	48.7%	63.3%	48.3%	64.0%
Sub5	100.0%	99.9%	50.2%	97.3%	99.9%	99.3%	99.7%	49.7%	97.3%	54.5%	100.0%

## Data Availability

The data used to support the findings of this study are not publicly available due to technology policy of Tongji University but are available from the corresponding author upon reasonable request.

## Supplementary Materials

Supplementary Materials The supplementary material contains the code of the algorithm in the manuscript. The detailed description of the code can be seen in the README.md file in the RAR file
